# The effects of migration on the practice and perception Female Genital Cutting (FGC) among the Horn of Africa's immigrants in Melbourne Australia

**DOI:** 10.3934/publichealth.2019.1.67

**Published:** 2019-02-26

**Authors:** Sadia Hassanen, Dawit Okubatsion Woldu, Rahma Mkuu

**Affiliations:** 1 Research Scientist, Department of Social Anthropology, Stockholm University, Sweden; 2 Assistant Professor, University of Houston-Clear Lake, College of Human Sciences and Humanities, Anthropology and Cross-cultural Studies, USA; 3 Department of Health and Kinesiology, Texas A&M University, USA

**Keywords:** Female Genital Cutting, culture, gender, immigration, Horn of Africa

## Abstract

This research examines the effects of migration on the practice and perception of Female Genital Mutilation or Cutting (FGM/C) among Horn of Africa immigrants in Melbourne Australia. According to UN 2016 report, on (FGM/C), there are at least 200 million girls and women alive today globally that have undergone some of form of FGM/C. The same report highlights that most of these practices are concentrated in parts of Africa, Middle East and South Asia. Our research employed in-depth semi-structured interviews with 50 men and women informants and five focus groups among the Horn of Africa immigrants living in Melbourne Australia. Interview and focus group data were analysed using MAXQUDA text analysis software to see emerging themes from the data. Upon the examination of the interviews and focus group data, we found that gender and immigration were the two factors that influenced immigrant's perception about FGC. Understanding the social and cultural dynamics on the perception of FGC among immigrant communities in the West could help in devising appropriate interventions to tackle FGC in several groups where this practice is commonly occurring.

## 1. Introduction

This study examines the impact of migration/immigration, gender, and cultural perceptions on the practice of Female Genital Cutting (FGC) among Horn of Africa migrants in Melbourne, Australia. We are using FGC as broader term to include the different kinds and levels of female genital modification and excisions practiced in the target populations. Furthermore, FGM is only one aspect of genital modification and does not include the different variations that exist among the diverse ethnic groups in the Horn of Africa. More importantly, FGM is a disempowering concept for victims and many of our informants deliberately avoided using FGM to describe their experience and instead use the term “Circumcision”. Finally, the use of the term could alienate governments and communities resulting in refusal to collaborate with researchers and public health officials. The Horn of Africa encompasses Somalia, Ethiopia, Eritrea, Djibouti, and Sudan. We selected these populations because of their similar social and cultural representations compared to other African migrants in Melbourne, Australia. Second, the Horn of Africa is one of the most conflicted zones in the African continent thus produces a diverse group of refugees and migrants. The refugees and migrants encompassed people from different walks of life, ethnicities, and religions seeking a safe place in the developed world including Australia. While immigrant communities originating from the Horn of Africa have distinct similarities and some interaction, they live disparate and separate lives. The migrants, much the same way they lived in their respective countries of origin, practice their traditions and hold on to cultural values they grew up with. In fact, most of these populations continue to interact with their communities in their respective countries, as geographic, political, and economic barriers are now no longer limited due to technological advances.

International migration is a concept that covers all types of migrations taking place across the border of a nation state [1],[2]. This concept is complicated because it refers to the politically recognized borders. Most of the migrants from the Horn of Africa are forced migrants that left their home countries because of violence and war. Forced migration is a process that may have a lifetime effect on the individual migrant/refugee in terms of economic, political, social, health, family relations, and wellbeing [3]–[7]. This type of displacement affects the sending/origin countries in a multitude of ways, such as loss of qualified professionals, deflating labour market, and deteriorating social value etc. [5]. Immigration also affects receiving countries. If the number of refugees is sizable, competition for social and economic infrastructures, such as employment, housing, school placement, healthcare services may increase [8]–[11]. In the worst-case scenario, this may lead to a conflict between refugees and host populations [3],[12]. Although the migrants from the Horn of Africa originate from different countries, they are similar culturally speaking as they typically share similar beliefs, values and traditions such as the practice of FGC. In as much as people flee due to multitude of reasons, the reason why people may stay in one area may be influenced by finding people who are culturally and socially similar to them [10]. The maintenance of culture and social identity is one of the most significant needs of many immigrants in the west. Migration is selective and each migrant's motive to move or to stay should be analysed individually taking into account the structural and cultural conditions that influence them [3] Examining the social and cultural need of the individual should not be viewed as isolated from the group that one identifies with and the broader social environment the person lives.

Therefore, exploring how the migration process affects the cultural and social life of migrant communities provides an important platform to understand the overall quality of life of migrants. Moreover, it may lead to understanding how people address or adapt with incompatible cultural practices such as FGC within their host country. Furthermore, understanding the adaptive process of cultural and traditional practices allows for the possibility of proposing interventions in both migrant communities and their countries of origin.

### 1.1. Female Genital Cutting

Female genital Cutting (FGC) is a worldwide problem practiced by different cultures and religions with special concentration in Asia, Middle East, and Africa. According to UN 2016 report on Female Genital Cutting/Mutilation (FGC/FGM), there are at least 200 million girls and women alive today globally that have undergone some of form of FGM. The same report highlights that most of these practices are concentrated in parts of Africa, Middle East and South Asia. The practice of FGC is cultural and includes all procedures that involve partial or total removal of the external female genitalia for non-medical reasons [13]. However, recent DHS data analysis from several countries in Africa and the Middle East shows a general decline trend on the prevalence of FGM between the 1990 and 2017 [14]. The same report acknowledges the decline trend in FGM varied greatly between countries and regions. The decline is attributed to concerted effort by several stakeholders that includes governmental agencies, local communities, religious leaders, academics and non-governmental agencies.

The WHO classifies FGC into four major categories based on severity and degree of invasiveness [15]–[17]. Type I includes partial and total removal of the clitoris known as *Clitoridectomy*. This type also could include the removal of the prepuce. Type II involves the partial or total removal of the clitoris and the labia *minora*, with or without excision of the *labia majora* and is known as Excision. Type III known as Infibulation, involves the narrowing of the vaginal opening through the creation of a covering seal and leaving a small hole for urine and menstrual blood to pass. Type IV involves all other harmful procedures to the female genitalia for non-medical purposes, such as pricking, piercing, incising, scraping and cauterizing the genital area.

The practice of FGM has been well-documented to impact both short and long term health of women [17],[18]. Short term health complications include; infections, hemorrhage, urinary pain, sepsis and increased risk to sexually transmitted infectious diseases during sexual intercourse and use of unsterilized traditional surgical instruments. The long term health implications of FGM include complications during birth such as high postpartum hemorrhage, increased risk of infant mortality, and increased length of maternal hospitalization [13],[17],[19]. Victims of FGC suffer from a long term permanent and irreversible tissue damage that have major negative implications on the health of girls throughout their life span [15],[18]. The pain from the scar and the overall experience of the practice could also have significant psychosocial consequences that will have major implications in the sexual and romantic relationships of the victims. FGC/M is a harmful traditional practice, which violates human right of the individuals. FGC/M should be considered one of the most pressing human right violations and public health problems that hinders women to live a dignified life.

While most of the different types of FGC are performed by traditional practitioners without anesthesia, about 18% of all FGC are done by western medical doctors in a hospital settings [13]. As a result, the WHO in collaboration with nine other UN partners published a report in 2010 labelling this practice as “medicalization” of FGC and concluded that the practice may have major implications to communities that immigrated from countries where these practices are very common. The medical procedures of FGC could be easily used by countries where FGC is very common or be utilized by medical professionals in western countries on immigrants from communities who practice FGC. While there has been an international uproar and condemnation of the practice from international organizations and United Nations bodies since 1994, the practice continues and results in immense suffering, lifelong health consequences, and even death for millions of women around the world [20].

Research pertaining to the practice and perception about FGC in immigrant communities in Europe, Australia and the US is very limited. A recent study conducted in Australia reports that 59 children were identified by pediatricians, as having undergone FGC, and 89.7% of the children were refugees born in Africa [21]. Most of the current literature about FGC in the western world focuses on the psychosocial consequences of FGC and the general experiences of women who had FGC in their country of origin and now live in the west [17],[22]–[24]. With the exception of [15] which heavily focused on a cross-sectional survey study about the perception of FGC among the Somalis in Norway, the existing literature on this subject mostly focuses on the health and human right implications of this culturally rooted practice. FGC is illegal in most western countries and people who are found guilty of committing FGC are persecuted. However, the enforcement of these laws are very rare and the legal definition/framework of this crimes remain unclear [20],[25]. Furthermore, efforts of these countries to work on the awareness of the cultural practice with immigrant communities are minimal.

Well-grounded and ethnographically based understanding of the perception and practice of FGC among immigrant community is critical. Furthermore, the impact of the shift in gender roles among these immigrant communities is crucial not only to understand the cultural complexity of the practice but also to devise a sensible cross-cultural intervention policy among both immigrant communities and their home countries.

### 2. Research method

This research employs qualitative research approaches with special focus on life histories and narratives. We collected in-depth unstructured and semi-structured interviews [26] and life histories to understand perspectives on FGC among immigrants from the Horn of Africa both before and after they arrived in Australia.

We conducted a systematic participant observation and five focus group discussions during the summer between May 2009 and September 2015. In addition, we conducted in-depth and semi-structured individual interviews, with 50 informants, using some aspects of Wengraf's Biographic Narrative Interpretive Method (BNIM) [27]. The method was constructed on the assumption that individuals are holistically constituted in cultural, social, and individual experiences. Consciously or unconsciously, these contexts and experiences in their histories form the background of the reflexive contention with everyday actions and interpretations.

The BNIM data collection style was important because the manner of expression and the informant's choice of words are important in interpreting the research data. Thus, language was important in this research since theoretical generalisation will require recognising supraliminal meanings of the individual informant's ideas about the study subject, and to some degree identifying his/her subliminal meanings through their choice of words and body language. A systematic interpretation of BNIM data of how life after migrating to Australia is expressed, enabled us to interpret the cultural/ideological meanings in which the act is located. Ideology and critical meanings in action [28] is an important interpretative tool in teasing out and navigating the spaces between the informants' own experiences and the cultural norm and values that they practise in their countries of origin and their current state of being in a different cultural setting.

The 50 informants, for the semi-structured interview, were composed of 25 women and 25 men, between the ages of 20 to 65 who migrated to Australia as adults from the Horn of Africa. Due to sensitivity of the topic as well as in order to protect the sample from being identified, we did not ask for country of origin. The first author travelled to Melbourne, Australia, every summer during the research period to conduct the interviews and stayed in the neighbourhood between 2—4 months where most of the immigrants live. We also conducted five focus groups that included two women only focus groups, 2 men only focus groups and one mixed focus group. Each focus group consisted of six participants. The mixed group had three women and three men participants.

Snowball sampling was used to recruit participants. Inclusion criteria for participants included being an immigrant from the Horn of Africa, and above the age 18. At least 12 of the women informants were interviewed more than one time between May 2009 and June 2015 to see how their perceptions on FGC has changed between 2009 and 2015. All interviews and discussions were held in places where informants felt free to speak about FGC.

The interviews were conducted with semi-structured open-ended questions intended to elicit or prompt a narration of FGC and other cultural issues participants considered important to their lives—a biography. Participants’ interviews begun by asking about broad cultural practices and then narrowed to discussing FGC. The main goal of this unstructured question is to start up a conversation and slowly narrow the discussion to FGC and other related cultural practices. Furthermore, the aim was also to pursue the method’s subsequent benefit of being able to add questions as they arise from the interviewees’ narrative. Only at the third stage of the method were the specific questions of the research posed to interviewees. The advantage of this method was that it would enable the researcher to have rapport with interviewees resulting in generation of more information about the impact of migration to Australia on the perception of FGC. Interviews were conducted in several languages, such as Tigre, Blien, Tigrinya, Arabic, Amharic, and English that are spoken in the Horn of Africa.

Our studies, strictly adhered to the general research guidelines of ethics agreed upon by the Swedish authorities (see www.vr.se and www.epn.se) and the University of Stockholm. All interviews were voluntary and participants were given the opportunity to discontinue their interview during the interview process. We guaranteed participants' confidentiality and protection of their information through the anonymization of information in accordance with the ethical guidelines.

### 2.1. Analyses

The semi-structured and focus group interviews were tape-recorded and subsequently transcribed. Transcribed text was coded and analyzed by MAXQUDA (Version 11), a text analysis software. Our analysis of the data focused on identifying some emerging themes and patterns among informants with regards to their perception of FGC. Thus, thematic analysis was used to see a patterned response in the data concerning what influences the change in perception and possibly practice of FGC among Horn of Africa informants. Our main goal was to understand informants' interpretation and their experience as a member of their cultural group and how migration to Australia and their contemporary context impacts their understanding of FGC.

To this end, the aim of this study was to understand how and in which ways migration affects perceptions about FGC, and whether migrating to Australia influenced our participants' attitudes and perspectives on FGC. The study further explored if there is any gender differences on the perception and attitudes toward FGC.

## 3. Results

Our analysis of both the participant observation and semi-structured interview data produced general patterns and themes. The semi-structured interviews results produced two themes with regards to perceptions and practice of FGC among Horn of Africa migrants living in Australia. The two themes were, 1) gender based experiences influences perceptions of FGC, 2), and immigration to Australia influenced perceptions and practice of FGC. Both the focus group data and participant observation data also confirm to the validity of these emergent themes from the semi-structured interviews.

Therefore, the themes demonstrate the influence of migration on change in power structure within the household, legal protections, and access to new ideas and values from the host country. Immigration, gender based experience/power shift in the household, and access to information about FGC impacted the changes in attitudes, perceptions, and practice of FGC among these immigrants.

### 3.1. Gender

Gender emerged as an important theme that influenced the perceptions of FGC as a cultural practice. The results from our study shows that there were clear differences between men and women with regards to the perception of FGC. Out of the 25 women we interviewed 23, strongly felt that FGC is a harmful cultural practice that needs to be stopped.

“To be honest I never discussed Female circumcision with my family or my daughters, now they are protected by law here in Australia and that is what matters to me, I am outside my country and don't want even imagine what could have happened to them if they were born and raised there” (Female informant).

The other two women felt that FGC is an important part of their culture that needs to be respected. Similarly 23 of the women felt that immigration to Australia helped them change their perception about the practice.

“What I want to take to Africa with me is what I am learning in this society (host country) about human rights and democracy. This consciousness will help me and other sisters to fight all the negative part of custom such as Female circumcision using education and empowering women” (Female informant).

The twelve women who were interviewed more than 2 times during the course of the research indicated that their perception and interest about FGC and voicing their opposition to this cultural practice has changed since they came to Australia. However, they expressed hesitation to speak about the practice with people who have established little rapport with them. Even though they knew it was a harmful practice when they were back in the respective home countries, but they wouldn't be able to speak up as freely about it as they do now. The women shared that the cultural pressure in their home countries silenced their opposition to FGC practice.

“In this country (Australia) women are better empowered and do not have any cultural pressure, now it is time for us to show an effort to eradicate this harmful practice. It will take time but hopefully it will end” (Female informant)

On the other hand, out of the 25 men, 19 of them believed that FGC is a cultural practice that is performed by older women as part of their culture and identity. The rest of the men felt that FGC is a woman's issue and the focus should be whether women wanted this practice or not.

“In our country women perform this practice, Men are passive about this issue, because they think it is women`s domain and role” (Male informant).

The 18 men interviewed expressed that they pay less attention to the problem even though their perspective to the practice has been evolving slowly over the years. Seven men however, shared their opposition to the practice;

“When I was in my country of origin I had seen and witnessed many women die but I didn't knew that heir death can be due to the complications caused by FGC. …It is an ancient culture which has to be eliminated but the problem is that people in my country of origin still understand FGC as a custom, which gives them identity, rather than as a harmful tradition. I was against all harmful traditions when I was in my country of origin, and also at present. I didn't liked tattoos, and other traditional medicine rituals which can cause infections and complications, but nobody listened to me, not even my family” (Male informant).

In general, the men who opposed the practice felt they have very little control to change the situation because they viewed the practice as a women's issue and that women are the ones who should fix it.

“One believe that women do this because it is necessary, what I mean is that women are mothers, sister, wives. Our society value and respect old people like (grandparents) opinion. Maybe this is why they don`t question the opinion of the old women, they see them as cultural and individual protectors. In the case of circumcision one should question their opinion and practice, this is how I think about it” (Male informant).

As illuminated above, moving from one place to another means questioning existing ideas, practice, evaluating norm, and values that root harmful practices like FGC. This seems the overriding theme in most of the women and some men from the Horn of African Diaspora in Australia.

### 3.2. Migration

Our study showed that most informants believed that their migration or immigration history influenced their views on FGC or at least provided them with an alternative perspective to the practice. Our general assessment from interviews showed that before migration 70% of women and 90% of men informants did not critically evaluate or have thoughts about the practice.

“I grew up in my country of origin and I had seen people preparing a big ceremony for such events (FGC). At that time, I did not know what I know today, and nobody had told me how harmful FGC is until I migrated to Australia. In Australia I saw a video about FGC and since then I began to think that FGC is harmful” (Male informant).

Most of interviewed individuals had left their respective countries in late 70's and mid 80's. Informants reported that exposure to different ideas and easily accessible information about the dangers of FGC in Australia has affected the way they view FGC. Additionally, the legal consequences of engaging in FGC also influenced their view on the matter. All of the women interviewed expressed the freedom to speak against FGC and the freedom to express their views among their peers without consequences provided them with opportunities to evaluate FGC more objectively. Being in Australia has given the women a chance to honestly share how they feel about FGC and the opportunity to speak to their daughters freely about their experience going through FGC in their respective countries. Furthermore, in Australia, the women have more access to accurate information about the effects of the practice and knowledge is not filtered through their husbands or other male guardians.

All interviewed women in this study shared that they had gone through FGC. The women though did not remember their experience of FGC as they were young when circumcised. Most shared that they were about two or three weeks old when they underwent circumcision. Eighty eight percent of interviewed women shared that they had reservations against the practice, for different reasons.

Some of the respondents felt that they are not directly affected by the practice, as they shared that they do not have daughters.

“Because I don't have any daughters I don't need to worry about FGC, and for my grandchildren they don't live here in Australia and since they are far away I cannot decided for them. It is up to their parents” (Female informant).

Some of the women gave health reasons and the rest were against it because of their shifted convictions and moral values. For example, some shared how their migration to Australia has changed their beliefs of FGC.

“I have one daughter and she was already circumcised before we came to Australia. I don't have any comment because I don't have another daughter. I think FGC is a harmful tradition and I am against it. My ideas against FGC are even stronger since I migrated to Australia” (Female informant).

One woman who has undergone the practice herself shared that she is not planning to subject her daughters to the tradition because she was able to learn many harmful things about the practice when she came to Australia;

“We, me and my husband, are against this horrible custom! During my wedding night I suffered a lot, I don't want my daughters to go through the same problem. Both my daughters are born in Australia and non-of them are going to be subjected to FGC. FGC is harmful for children and dangerous for women's health. Coming to Australia was an eye opening for me and my family about this practice” (Female informant).

In addition, the focus group interviews produced both gender and immigration as the influencing factors to changes on FGC perception among immigrants in Australia. All participants in the focus group agree changes in gender dynamics has influenced the changes in perception about FGC among horn of African diaspora communities in Australia. While most of the men focus group feel negative or neutral about the changes in perception about FGC, almost all of the women focus group participants feel the change in perception due to migration are as positive.

## 4. Discussion

The results of our research reflect the broader social and cultural understanding of FGC among Horn of Africa immigrants living in Australia. The perceptions of the practice of FGC were largely defined along gender lines and social expectations. Cultures in the Horn of Africa are in many ways considered as patriarchal and traditional societies. Due to gendered nature of several social and cultural expectations, FGC perception and understanding was very much influenced by gender roles and gender expectation among these diaspora community. Women perceived FGC as culturally imposed harmful practice while men mainly viewed as a women's issue. The men in our study felt culturally excluded from FGC and thus distanced themselves from having limited involvement in the practice. Our results are supported by other studies [29]–[32] that have similar conclusions. Similar to other cultural practices such as the bodily modifications that are imposed on women, FGC is a cultural and traditional practice that is generally unquestioned among women and men in many cultures[31],[32].

In addition to being a practice that is associated with expectations of how women should be presented, FGC is also associated with power structural dynamics [32]. This cultural practice is maintained by the power structure in the society, which highly favours men. For example, if a woman questions FGC, as a bad practice, she could be ostracized by society or face punishment. Women in our study shared that being in Australia gave them the opportunity to both question and challenge the practice without having to experience or fear implications from their families or general society. The consequences of straying away from cultural traditions and practices such as FGC are often far more damaging to a woman compared to a man. These findings are supported by previous literature reporting that women fear consequences or being stigmatized from straying away from traditional practices, especially in the absence of education opportunity and lack of empowerment [18],[33]. Furthermore, these patriarchal dominated societies create a cultural norm that FGC is an important initiation rite any young girl should go through in order to be a socially accepted woman in society. This initiation rite therefore is viewed not only as a patriarchal practice of male dominance but also as an experience of womanhood thus has been passed down as a practice that has come to be accepted and valued by women themselves [34]. As tradition gatekeepers, women have come to embrace the practice despite the dire consequences to their health and lives [34].

Migration as factor of influencing perceptions of FGC also emerged as a theme in our study, migration brought a new gender and value dynamics in diaspora population living in the west. As expressed by participants in the study, living in Australia has exposed them to different ideals, values, and knowledge on implications of practices such as FGC. Living in a new culture with different gender dynamics has influenced the way the participants view the practice. Many participants shared that they are now exposed to information of the consequences of FGC and that they feel freer to discuss the practice in Australia compared to their countries of origin. Participants also shared that they do not wish to have their daughters undergo the practice, aligning with findings from other studies among immigrants living in Western countries[35],[36]. That being said, FGC is not restricted to the Horn of Africa. Besides, FGC and women's oppression is a universal problem, since women in all parts of the world are struggling to overcome women's subordination[37],[38].

According to Hondagenu (1994) Mexican immigrant women in the United States regain some form of power from the patriarchal system in their home country. Both men and women bring money to the household that enabled Mexican immigrant women to challenge their husbands and partners. These gender dynamics are the result of immigration to a more liberal society where women's rights are more respected and women can also accumulate power through economic opportunity in their host countries that wasn't necessary the case in Mexico [39].

Based on our study immigration, shift in gender relations, access to new information and ideas have contributed to changes in the perception of FGC among migrants from the Horn of Africa. Our study suggests that it is imperative to note that empowerment of women in countries of origin in conjugation with public education and working with local communities could have a positive impact. The inclusion of religious leaders and other key figures with high cultural and social capital could play a positive role to address the FGC issues many countries face both in the Horn of Africa and beyond.

## 5. Conclusion

Female genital mutilation or circumcision is a challenging cultural practice that has resisted several government efforts and legislations in many countries to eradicate it. Our research does not provide the ultimate solution to the problem; however, it contributes to the current literature adding contextual understanding of the perception of FGC among the target population. The main contribution of our work is that we explored FGC by going beyond examining cultural construction of it. We sought to explore how different social, political, and cultural environment influence the perception and practice of FGC. Specifically, we sought to understand the social, political, and cultural forces that played out on immigrants, who came from countries where this practice is common to change their perspective on FGC. Applying those cultural and social changes in countries where this practice is prevalent could mitigate the problem and save millions of girls from lifelong health and psychological suffering. Developing sustainable women's empowerment programs, engaging men in the conversations, and introducing the negative impact of FGM in school curriculums could accelerate changes on society's perspective to FGC.

## References

[1] Tomescu IR (2013). International Migration: Security Implications. Res Sci Today.

[2] Al-Sharmani M (2004). Refugee livelihoods: Livelihood and diasporic identity constructions of Somali refugees in Cairo: UNHCR, Evaluation and Policy Analysis Unit.

[3] Westin C (1999). Regional analysis of refugee movements: Origins and response. Refugees: Perspectives on the experience of forced migration.

[4] Hassanen S, Westin C, Olsson E (2013). People on the move: experiences of forced migration with examples from various parts of the world.

[5] Flahaux ML, De Haas H (2016). African migration: trends, patterns, drivers. Comp Migr Stud.

[6] Crisp J (2000). Africa's refugees: patterns, problems and policy challenges. J Contemp African Stud.

[7] Al-Sharmani M (2004). The American University in Cairo, Forced Migration and Refugee Studies Program (FMRS) Working Paper. Livelihood and diasporic identity constructions of Somali refugees in Cairo.

[8] Spiegel PB, Checchi F, Colombo S (2010). Health-care needs of people affected by conflict: future trends and changing frameworks. Lancet.

[9] Simich L, Beiser M, Stewart M (2005). Providing social support for immigrants and refugees in Canada: Challenges and directions. J Immigr Health.

[10] Gordon JA, Liu X (2015). Bridging home and host country: educational predispositions of Chinese and Indian recent immigrant families. Int J Multicult Educ.

[11] Grove NJ, Zwi AB (2006). Our health and theirs: forced migration, othering, and public health. Soc Sci Med.

[12] Rabrenovic G (2007). When hate comes to town: Community response to violence against immigrants. Am Behav Sci.

[13] Organization WH, UNICEF, Association WM (2010). Global strategy to stop health-care providers from performing female genital mutiliation.

[14] Kandala NB, Ezejimofor MC, Uthman OA (2018). Secular trends in the prevalence of female genital mutilation/cutting among girls: a systematic analysis. BMJ Glob Health.

[15] Gele AA, Johansen EB, Sundby J (2012). When female circumcision comes to the West: Attitudes toward the practice among Somali Immigrants in Oslo. BMC Public Health.

[16] Organization WH (2012). Understanding and addressing violence against women: intimate partner violence.

[17] Suardi E, Mishkin A, Henderson SW (2010). Female genital mutilation in a young refugee: A case report and review. J Child Adolesc Trauma.

[18] Gruenbaum E (2001). The female circumcision controversy: an anthropological perspective.

[19] Hock RR (2007). Human sexuality: Pearson/Prentice Hall.

[20] Naughton L, D'Alessio A (2015). Invisible backbone. Community Pract.

[21] Zurynski Y, Phu A, Sureshkumar P (2017). Female genital mutilation in children presenting to Australian paediatricians. Arch Dis Child.

[22] Kaplan-Marcusan A, Torán-Monserrat P, Moreno-Navarro J (2009). Perception of primary health professionals about female genital mutilation: from healthcare to intercultural competence. BMC Health Serv Res.

[23] Ogunsiji OO, Wilkes L, Jackson D (2007). Female genital mutilation: Origin, beliefs, prevalence and implications for health care workers caring for immigrant women in Australia. Contemp Nurse.

[24] Mabilia M (2013). FGM or FGMo? Cross-cultural dialogue in an Italian minefield. Anthropol Today.

[25] La Barbera MC (2017). Ban without prosecution, conviction without punishment, and circumcision without cutting: a critical appraisal of anti-FGM laws in Europe. Global Jurist.

[26] Bernard HR (2012). Social research methods: Qualitative and quantitative approaches.

[27] Wengraf T (2001). Biographic narrative and semi-structured methods, *Qualitative research interviewing*, SAGE Research Methods.

[28] Boréus K, Bergström G (2012). Textens mening och makt: metodbok i samhällsvetenskaplig text-och diskursanalys.

[29] Akinsulure-Smith AM, Chu T (2017). Knowledge and attitudes toward female genital cutting among West African male immigrants in New York City. Health Care Women Int.

[30] Vogt S, Efferson C, Fehr E (2017). The risk of female genital cutting in Europe: comparing immigrant attitudes toward uncut girls with attitudes in a practicing country. SSM Popul Health.

[31] Hansen P (2008). Circumcising migration: Gendering return migration among Somalilanders. J Ethnic Migr Stud.

[32] Vissandjée B, Kantiébo M, Levine A (2003). The cultural context of gender, identity: female genital, excision and infibulation. Health Care Women Int.

[33] Van Rossem R, Meekers D, Gage AJ (2015). Women's position and attitudes towards female genital mutilation in Egypt: A secondary analysis of the Egypt demographic and health surveys, 1995–2014. BMC Public Health.

[34] Organization WH (2008). Eliminating female genital mutilation: an interagency statement-OHCHR, UNAIDS, UNDP, UNECA, UNESCO, UNFPA, UNHCR, UNICEF, UNIFEM.

[35] Koukoui S, Hassan G, Guzder J (2017). The mothering experience of women with FGM/C raising ‘uncut’daughters, in Ivory Coast and in Canada. Reprod Health.

[36] Johansen REB (2017). Undoing female genital cutting: perceptions and experiences of infibulation, defibulation and virginity among Somali and Sudanese migrants in Norway. Cult Health Sex.

[37] Diaz JA (1993). Choosing integration: a theoretical and empirical study of the immigrant integration in Sweden.

[38] El Dareer A (1982). Woman, why do you weep? Circumcision and its consequences.

[39] Hondagneu-Sotelo P (1994). Gendered transitions: Mexican experiences of immigration.

